# Inhibiting H3K27 Demethylases Downregulates CREB‐CREBBP, Overcoming Resistance in Relapsed Acute Lymphoblastic Leukemia

**DOI:** 10.1002/cam4.70596

**Published:** 2025-01-10

**Authors:** Juan Lazaro‐Navarro, Clara Alcon, Mathurin Dorel, Lina Alasfar, Lorenz Bastian, Claudia Baldus, Kathy Astrahantseff, Marie‐Laure Yaspo, Joan Montero, Cornelia Eckert

**Affiliations:** ^1^ Department of Pediatric Oncology/Hematology Charité‐Universitätsmedizin Berlin Berlin Germany; ^2^ German Cancer Consortium (DKTK) German Cancer Research Center (DKFZ) Heidelberg Germany; ^3^ Department of Biomedical Sciences, Faculty of Medicine and Health Sciences University of Barcelona Barcelona Spain; ^4^ Networking Biomedical Research Center in Bioengineering, Biomaterials and Nanomedicine (CIBER‐BBN) Madrid Spain; ^5^ Max Planck Institute for Molecular Genetics Berlin Germany; ^6^ Medical Department II, Hematology/Oncology University Medical Center Schleswig‐Holstein Campus Kiel Germany

**Keywords:** apoptosis, drug discovery and delivery, epigenetics, leukemia

## Abstract

**Background:**

CREB binding protein (CREBBP) is a key epigenetic regulator, altered in a fifth of relapsed cases of acute lymphoblastic leukemia (ALL). Selectively targeting epigenetic signaling may be an effective novel therapeutic approach to overcome drug resistance. Anti‐tumor effects have previously been demonstrated for GSK‐J4, a selective H3K27 histone demethylase inhibitor, in several animal models of cancers.

**Methods:**

To characterize the effect of GSK‐J4, drug response profiling, CRISPR‐Dropout Screening, BH3 profiling and immunoblotting were carried out in ALL cell lines or patient derived samples.

**Results:**

Here we provide evidence that GSK‐J4 downregulates cyclic AMP‐responsive element‐binding protein (CREB) and CREBBP in B‐cell precursor‐ALL cell lines and patient samples. High CREBBP expression in BCP‐ALL cell lines correlated with high GSK‐J4 sensitivity and low dexamethasone sensitivity. GSK‐J4 treatment also induced Bcl‐2 and Bcl‐XL dependency and apoptosis.

**Conclusions:**

This study proposes H3K27 demethylase inhibition as a potential treatment strategy for patients with treatment‐resistant ALL, using CREBBP as a biomarker for drug response and combining GSK‐J4 with venetoclax and navitoclax as synergistic partners.

## Graphical abstract

This study investigates the effect of epigenetic regulator GSK‐J4 in the downregulation of CREB and CREBBP in acute lymphoblastic leukemia (ALL) cell lines and relapsed patient samples, leading to apoptosis induction and cell cycle arrest. We propose H3K27 demethylase inhibition as a potential treatment strategy for patients with treatment‐resistant ALL, using CREBBP as a biomarker for drug response and combining GSK‐J4 with venetoclax and navitoclax as synergistic partners.

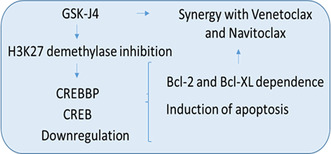

## 1 Introduction

Implementing contemporary treatment concepts has raised relapse‐free survival to > 80% in children with acute lymphoblastic leukemia (ALL) [1]. Despite significant progress in risk‐adapted treatment concepts, the emergence of drug resistance at relapse remains a major challenge to the successful treatment of ALL. At relapse, pediatric B‐cell precursor (BCP)‐ALL is enriched with somatic mutations in the genes for several epigenetic regulators, including the CREB binding protein (*CREBBP*), lysine demethylase 6A (*KDM6A*, previously known as *UTX*), lysine methyltransferase 2D (*KMT2D*) and SET domain containing 2, histone lysine methyltransferase (*SETD2*), suggesting the role of epigenetic regulators in development of treatment resistance [2, 3]. *CREBBP* alterations consisting of either sequence mutations or deletions occur in 18.3% of patients with relapsed ALL [2]. Dysregulation of the cAMP‐response element binding protein 1 (*CREB1*), is implicated in ALL drug resistance (e.g., to glucocorticoids) [4]. CREB1 is a transcription factor involved in epigenetic regulation of key epigenetic elements (e.g., enhancer of zeste 2 polycomb repressive complex 2 subunit; *EZH2*), and its overexpression leads to increased histone 3 lysine 27 trimethylation (H3K27me3) [5]. CREBBP forms complexes with CREB1 or E1A‐binding protein (EP300), whose histone acetyltransferase activity modulates cellular processes such as growth, survival, differentiation, and hematopoiesis, by regulating transcription factors such as MYC [6] and glucocorticoid responsive genes [4]. These findings suggest that targeting key epigenetic regulators in ALL [7] could be a new approach to for the treatment of relapsed ALL patients, and raises the need to investigate novel inhibitors in preclinical settings capable of selectively targeting epigenetic regulators that can overcome drug resistance.

## 2 Material and Methods

Detailed information on applied material and methods is provided in Appendix S1.

## 3 Results

**FIGURE 1:**
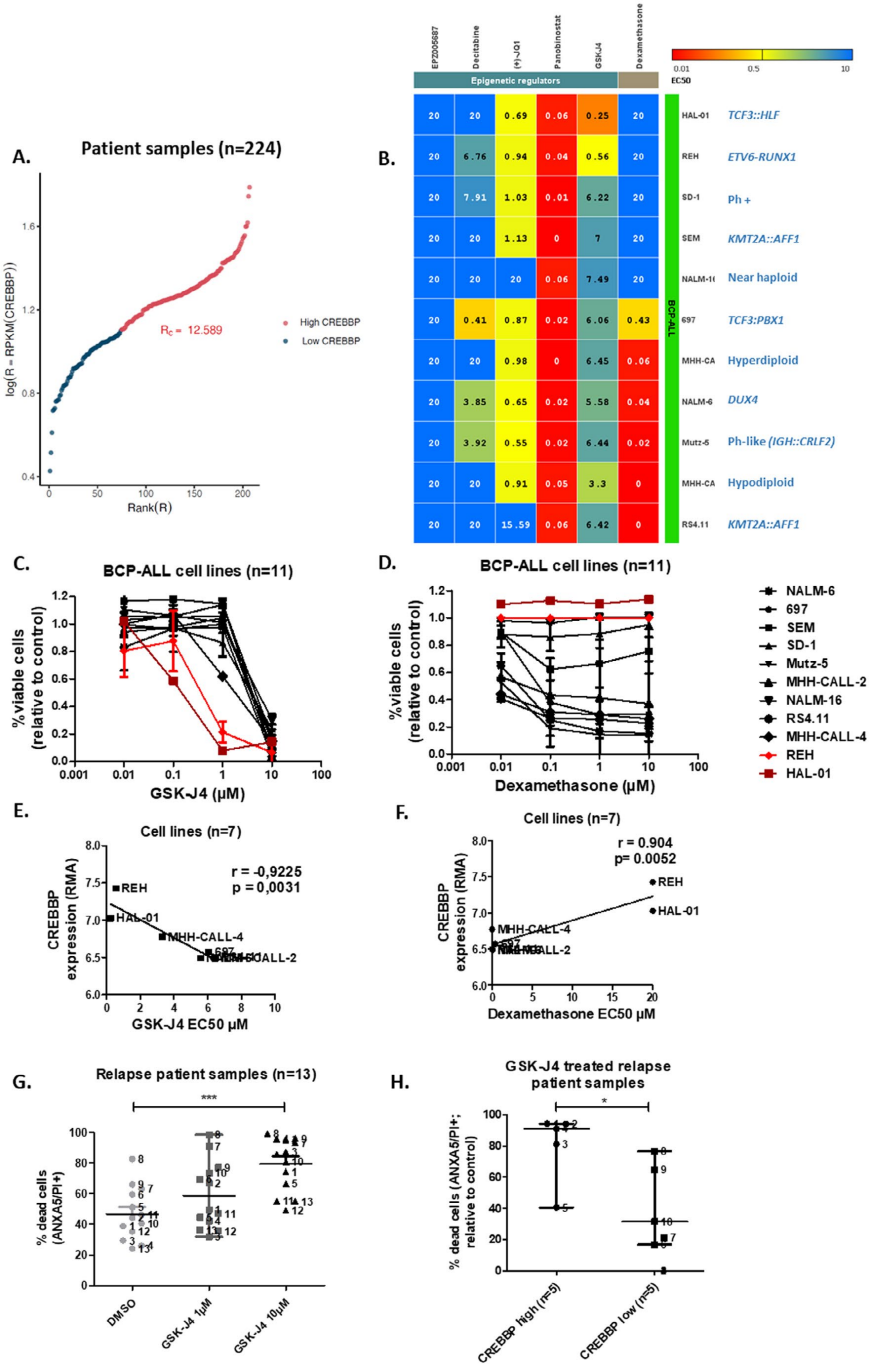
Targeted drug screen identified sensitivity to GSK‐J4 in dexamethasone resistant cell lines, which correlated with CREBBP gene expression levels in cell lines and relapse patient samples. (A) Heterogeneity of patient samples expressing CREBBP (*n* = 244). Samples overexpressing CREBBP were identified by ordering the samples by their expression and finding the cutoff expression (Rc) in which the Jaccard distance in the log Expression—Sample expression Rank is maximized, providing a bias‐free separation between low and high expressors of the genes. (B) Viability of BCP‐ALL cell lines (*n* = 11) was assessed by WST‐1 assay after incubation for 48 h with epigenetic regulators (*n* = 5). EC50 concentrations are shown (μM). Cell viability curves of BCP‐ALL cell lines (*n* = 11) measured with WST‐1 assay after 48 h treatment with serial dilutions of (C) GSK‐J4 and (D) dexamethasone. Figure shows mean and SD from *n* = 3 independent experiments conducted in triplicates, from same experiments as data shown in panel (A). (E) CREBBP gene expression is directly proportional to the sensitivity to GSK‐J4 in BCP‐ALL cell lines (*n* = 11) and (F) inverse proportional to the sensitivity to dexamethasone in BCP‐ALL cell lines (*n* = 7). Pearson correlation coefficient (*r*) and *p* values are shown. (G) Proportion of dead cells in primary ALL samples (*n* = 10), measured Proportion of dead cells in primary ALL samples treated with 1/10 μM GSK‐J4. After 48 h, ANXA5 and PI staining was detected by flow cytometry. (H) Proportion of dead cells in primary ALL samples (*n* = 10) from panel (G) where RNAseq data was available. Samples were clustered in RNA CREBBP high and low (≥ and < median 20, 2 Reads Per Kilobase per Million mapped reads, RPKM, respectively), after treatment with 10 μM GSK‐J4 and normalized to untreated control. Median and interquartile range (IQR) are shown. *p* values were calculated using Mann–Whitney *U* test. *p ≤ 0.05; ***p ≤ 0.001. Additional information on applied methods and samples is provided in Appendix S1.

RNAseq analysis in a cohort of 224 pediatric patients with relapsed BCP‐ALL identified heterogeneous CREBBP expression, with 19 relapses (8.5% of cases) overexpressing *CREBBP* (Figure 1A). *CREBBP* RNA levels correlated positively with *EP300* levels and negatively with *CREB1* (encoding CREB protein; Figure 1A, Figure S1) levels. To identify novel targetable vulnerabilities regulating the relapse ALL epigenome, a drug screen including 5 epigenetic regulators and dexamethasone was carried out in BCP‐ALL cell lines (*n* = 11, Figure 1B). Among the most compelling drug sensitivity patterns identified, was that the dexamethasone‐resistant BCP‐ALL cell lines, HAL‐01, and REH, (half‐maximal effective concentrations; EC50 > 20 μM) were the most sensitive to GSK‐J4 (EC50 = 0.25 μM and 0.56 μM, median EC50 = 6.2 μM, Figure 1C,D). GSK‐J4 selectively inhibits KDM6A and KDM6B H3K27 demethylases. GSK‐J4 EC50 values were significantly inversely correlated with *CREBBP* RNA expression in BCP‐ALL cell lines (*r* = −0.92, *p* = 0.003, Figure 1E). Dexamethasone EC50 values were significantly correlated with higher *CREBBP* RNA expression (*r* = 0.9, *p* = 0.005, Figure 1F), suggesting that cell lines expressing higher levels of *CREBBP* are less sensitive to dexamethasone but more sensitive to GSK‐J4. Treatment with GSK‐J4 reduced BCP‐ALL cells (*n* = 4 lines) at the cell cycle phase S/G2 transition and, although not significantly, enhanced the proportion of cells in G1, suggesting that GSK‐J4 treatment arrests the cell cycle at G1 (Figure S2). To better reflect biological and clinical heterogeneity in BCP‐ALL cases, patient‐derived samples (*n* = 13, patient information in Table S1), obtained after first or second relapse, were treated in vitro with GSK‐J4. GSK‐J4 significantly induced apoptosis (*p* = 0.0005) in patient‐derived BCP‐ALL cells (Annexin V and PI staining, Figure 1G). In accordance with our findings in cell lines, sensitivity to GSK‐J4 was significantly higher (59.6% median increase, *p* = 0.03) in patient‐derived samples expressing higher *CREBBP* RNA levels compared to patient samples with lower *CREBBP* levels (Figure 1H). Overall, our data suggest high *CREBBP* expression is a biomarker determining dexamethasone‐resistant BCP‐ALL cell sensitivity to GSK‐J4.

**FIGURE 2:**
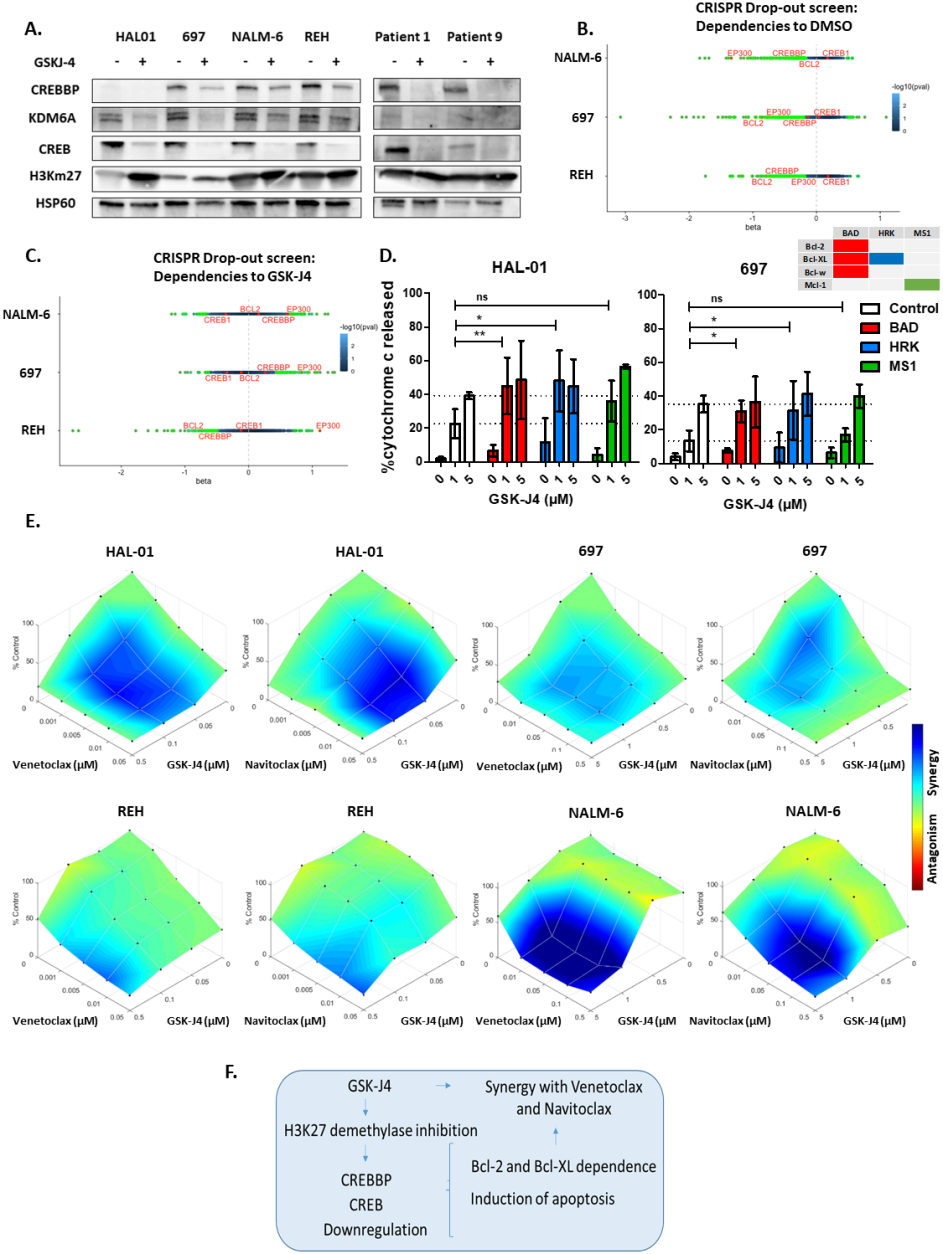
Molecular characterization of the implication of GSK‐J4 treatment and the CREB‐CREBBP complex, leading to the dependency of BCL2 and BCL‐xL signaling and synergy with BH3 mimetics. (A) Western blot analysis performed with the indicated antibodies, on lysates of BCP‐ALL cell lines (*n* = 4) and a primary sample treated for 24 h with vehicle (dimethylsulfoxide; DMSO) or 10 μM GSK‐J4. HSP60 served as the loading control. Dependencies to DMSO (B) or GSK‐J4 (C) were assessed in NALM‐6, 697, and REH cell lines expressing Cas9 and transfected with a CRISPR library [8]. Cells were treated with DMSO or GSK‐J4 (0.3 μM) for 14 days, followed by harvesting and cell count determination. Beta scores were calculated using MAGeCK MLE v0.5.9.5 (Li et al., 2014). Negative beta scores indicate gene dependency, while positive scores suggest enhanced survival. Each dot represents a gene, color‐coded according to the significance of dependency (−log10(*p*‐value)) using the accompanying scale. Genes with significant dependencies (*p* < 0.05) are highlighted in green. (D) BH3 profiling of BCP‐ALL cell lines HAL‐01 and 697 exposed (16 h) to GSK‐J4 (1‐5μM) followed by exposure to increasing concentrations of the inhibitor peptides BAD, HRK, MS1 or vehicle control (1 h) before cytochrome C release was measured by flow cytometry. *p* values were calculated using Mann–Whitney *U* test; ns: Not significant; *p ≤ 0.05; **p ≤ 0.01. (E) Combination effects on viability of BCP‐ALL cell lines HAL‐01, 697, REH and NALM‐6 were analyzed 48 h after treatment with serial dilutions of single and combined compounds (20 combined concentrations: Venetoclax/navitoclax alone (*n* = 4), GSK‐J4 alone (*n* = 3) and all combinations of these). 1:10 dilutions for each compound where applied to high sensitive REH and HAL‐01 cells. Combination effects were determined by HSA reference model (Combenefit software) integrating *n* = 3 independent experiments. Each data point represents one drug or combination. (F) Schematic representation of how H3K27 demethylase inhibitor GSK‐J4 leads to CREB‐CREBBP downregulation, followed by the induction of cell cycle arrest and apoptosis mediated by Bcl‐2 and Bcl‐XL dependence, leading to the synergy between GSK‐J4 and BH3 mimetics venetoclax and navitoclax. Additional information on applied methods and samples is provided in Appendix S1.

To study the interaction between GSK‐J4 treatment and the CREB1/CREBBP complex, western blot analysis was carried out in different BCP‐ALL cell lines (*n* = 4) and 2 patient‐derived samples. GSK‐J4 treatment downregulated CREB1 and KDM6A protein levels across the different ALL cells tested (Figure 2A, Figure S3). Except for HAL‐01, which is not detected by the CREBBP antibody because it harbors a *CREBBP* mutation (c.6299 T > G), all the BCP‐ALL cells demonstrated downregulated CREBBP protein levels in response to GSK‐J4 treatment (Figure 2A, Figure S3). To better understand the molecular mechanisms underlying GSK‐J4 sensitivity, we conducted a CRISPR drop‐out screen [8] in 697, NALM‐6, and REH cell lines expressing Cas9. A comparison between wild‐type and knockout cells revealed a dependency on *EP300* and *CREBBP* across all cell lines, as well as on *BCL2* (negative beta values; Figure 2B). Dependence to *CREBBP* was seen highest in GSK‐J4 sensitive cell line REH, compared to less sensitive cells NALM‐6 and 697. In GSK‐J4‐treated cells, *EP300* exhibited a protective effect (positive beta values, Figure 2C). Notably, in REH cells, which are sensitive to GSK‐J4, synthetic lethality was observed in GSK‐J4 treated cells harboring *CREBBP KO* and *BCL2 KO*. These findings further underscore the role of *CREBBP* in mediating GSK‐J4 sensitivity, identifying it as a critical signaling element, essential for ALL cell survival.

Dynamic BH3 profiling [9] is a functional assay allowing the study of underlying mechanisms of cellular adaptations to overcome apoptosis in different ALL cells before and after treatment [10]. We performed dynamic BH3 profiling in HAL01 and 697 cell lines treated with GSK‐J4 together with either BAD, HRK or MS1 peptides that respectively target the Bcl‐2/Bcl‐XL/Bcl‐w, Bcl‐XL only or Mcl‐1 anti‐apoptotic proteins. Treatment with BAD and HRK peptides but not MS1, significantly increased cytochrome C release after treatment with 1 μM GSK‐J4 in both cell lines (Figure 2D), indicating the cells are dependent on Bcl‐2 and BCL‐XL to resist apoptosis induced by GSK‐J4. This suggests a potential targetable vulnerability that could be overcome by combining GSK‐J4 with an appropriate BH3 mimetic drug. Consequently, we analyzed synergy between GSK‐J4 combined with either venetoclax (Bcl‐2 inhibitor) or navitoclax (Bcl‐2/Bcl‐XL inhibitor). Serial dilutions of these BH3 mimetics, 20 combined concentrations of venetoclax/navitoclax alone (*n* = 4), GSK‐J4 alone (*n* = 3) and all combinations of these, were applied to BCP‐ALL cell lines (*n* = 4). Using the HSA reference model, synergy was analyzed in 4 BCP‐ALL cell lines, identifying at least one significant synergistic combination in each compound and cell line tested (Figure 2E, Table S2). The synergy between BH3 mimetics and GSK‐J4 is strongest in the NALM‐6 cell line, which is least sensitive to GSK‐J4 alone, and weaker in the most sensitive cell line, REH (8 and 1–2 significant synergistic combinations respectively). Our results demonstrate that inhibiting H3K27me3 demethylases induces a Bcl‐2 and Bcl‐XL dependency in cell lines, both sensitive and resistant to GSK‐J4, leading to a synergistic interaction between GSK‐J4 and the BH3 mimetics, venetoclax, and navitoclax.

## 4 Discussion

Previous studies have reported the anti‐tumor effect of GSK‐J4 in xenograft mouse models of acute myeloid leukemia (AML) [11] and breast cancer [12], and for GSK‐J4 in combination with oxaliplatin in xenograft mouse models of colorectal cancer [13]. Higher *KDM6B* expression levels was linked to worse survival in diffuse large B‐cell lymphoma patients, and cell lines with mutations in *CREBBP/EP300* presented an increased sensitivity to GSK‐J4 treatment [14]. Here we propose a model in which GSK‐J4 treatment downregulates CREB and CREBBP expression in ALL cells, which subsequently induces cell cycle arrest and even apoptosis, with higher sensitivity seen in cells expressing high CREBBP levels. High *CREBBP* expression was also correlated with heightened GSK‐J4 sensitivity and diminished dexamethasone sensitivity in multiple ALL cell lines on RNA and protein levels in the recently published FORALL [15] cell line database (Figure S4). Our data is in accordance with previous results from AML cell lines, where CREB1 protein levels decreased upon GSK‐J4 treatment, a process regulated by CREB1 phosphorylation at the Ser133 residue by protein kinase A (PKA) and leading to its proteasomal degradation [16].

We provide evidence here that inhibiting H3K27me3 demethylases is a potential treatment strategy for relapse patients with ALL who do not respond to dexamethasone. We propose a strategy (Figure 2F) in which CREBBP expression can be used as a potential biomarker to indicate response to GSK‐J4, which induces a Bcl‐2 and Bcl‐XL dependency that can be targeted by combining GSK‐J4 with clinically available [17, 18] synergistic partners venetoclax and navitoclax.

## Author Contributions

**Juan Lazaro‐Navarro:** conceptualization (lead), data curation (lead), formal analysis (lead), investigation (lead), methodology (lead), writing – original draft (lead), writing – review and editing (lead). **Clara Alcon:** data curation (equal), formal analysis (equal), methodology (equal), writing – review and editing (equal). **Mathurin Dorel:** methodology (equal), writing – review and editing (equal). **Lina Alasfar:** data curation (equal), formal analysis (equal), writing – review and editing (equal). **Lorenz Bastian:** conceptualization (equal), funding acquisition (equal), supervision (equal). **Claudia Baldus:** funding acquisition (equal), supervision (equal), writing – review and editing (equal). **Kathy Astrahantseff:** writing – original draft (equal), writing – review and editing (lead). **Marie‐Laure Yaspo:** supervision (equal), writing – review and editing (equal). **Joan Montero:** funding acquisition (equal), methodology (equal), supervision (equal), writing – review and editing (equal). **Cornelia Eckert:** conceptualization (equal), funding acquisition (lead), project administration (lead), supervision (lead), writing – original draft (equal), writing – review and editing (equal).

## Ethics Statement

All patients were enrolled in the IntReALL SR/HR 2010 trials or the ALL‐REZ BFM registry, approved by the State Office for Health and Social Affiars Berlin and the Institutional Review Board of the Charité Universitätsmedizin Berlin, Berlin, Germany respectively (ClinicalTrials.gov identifier: NCT01802814/NCT03590171). Written informed consent was obtained from patients or guardians.

## Conflicts of Interest

J.M. is co‐inventor of dynamic BH3 profiling (patented by Dana‐Faber Cancer Institute) and has received royalties, was a paid consultant for Oncoheroes Biosciences and Vivid Biosciences, is an unpaid board member for The Society for Functional Precision Medicine, and he is currently collaborating with AstraZeneca. No potential conflicts of interest were disclosed by the other authors.

## Supporting information


Appendix S1.

## Data Availability

The data underlying this study will be made available upon request to the authors via email.
