# Supplementary material for: Inhibiting H3K27 Demethylases Downregulates CREB‐CREBBP, Overcoming Resistance in Relapsed Acute Lymphoblastic Leukemia
Source: Cancer Med. 2025 Jan 10;14(1):e70596. doi: 10.1002/cam4.70596 (PMC11719120; doi:10.1002/cam4.70596)
Supplement: Supplementary file 1 — Appendix S1. [file CAM4-14-e70596-s001.docx]

**Supporting Information**

The following supplementary files include:

Supplementary Material and Methods pages 2 - 4

Supplementary Figures 1-4 pages 5 - 8

Supplementary Tables 1-2 pages 9 - 10

**Reference**

19. Yang W, Soares J, Greninger P, et al. Genomics of Drug Sensitivity in Cancer (GDSC): A resource for therapeutic biomarker discovery in cancer cells. Nucleic Acids Res. 2013;41(D1):955-961. doi:10.1093/nar/gks1111

20. Tate JG, Bamford S, Jubb HC, et al. COSMIC: the Catalogue Of Somatic Mutations In Cancer. Nucleic Acids Res. 2019;47(D1):D941-D947. doi:10.1093/nar/gky1015

**Supplementary Material and Methods**

**Cell lines and patient primary samples**

Human BCP-ALL cell lines were purchased from the German Collection of Microorganisms and Cell cultures (DSMZ, Braunschweig, Germany). Cell lines were cultivated in RPMI (Gibco, Montana, United States) supplemented with 10% fetal calf serum and 5% penicillin-streptomycin at 37°C and 5% CO_2_. Cell lines were routinely tested for mycoplasma infection, the expression of the corresponding surface markers and fusion genes by flow cytometry, quantitative real-time PCR and FISH.

BCP-ALL primary samples were cultured in StemSpan™ SFEM II media (Stemcell, Vancouver, Canada) supplemented with 20ng/mL IL-3 and IL-7 (Stemcell, Vancouver, Canada). Samples were selected according to availability from viable frozen or fresh cells. All patients were enrolled in the *IntReALL SR/HR 2010* trials or the *ALL-REZ BFM* registry, approved by the *State Office for Health and Social Affiars Berlin* and the Institutional Review Board of the *Charité Universitätsmedizin Berlin*, Berlin, Germany respectively (ClinicalTrials.gov identifier: NCT01802814/NCT03590171). Written informed consent was obtained from patients or guardians.

**Viability, apoptosis and cell cycle analysis**

Viability and proliferation was quantified after treatment using the WST-1 assay for cell proliferation and viability (Roche Applied Science, Rotkreuz, Switzerland) according to manufacturer’s instructions. EC50 values were calculated from viability curves (generated using WST-1 assay data) using a linear regression model in Graphpad Software (v.7, San Diego, CA, USA). Corresponding drugs were applied in 4-5 concentrations (10/1/0.1/0.01/0.001 µM) to generate data to calculate EC50 values. EC50 heatmaps were generated using Gitools software (v2.3.1, Barcelona, Spain). Apoptosis was assessed by staining cells with Annexin A5 (Biolegend, San Diego, USA) and propidium iodide (PI; Miltenyi Biotec, North Rhine-Westphalia, Germany). Flow cytometry analysis used BD LSRFortessa™X-20 cell analyzer (BD Biosciences, San Diego USA) and FlowJo CL Software v.10 (Treestar, Ashland, USA). All inhibitors used in different treatment experiments were purchased from Selleckchem (Houston, USA). Cell cycle analysis was carried out flow cytometrically after staining cells with CytoPhase™ Violet (Biolegend, San Diego, USA) and PI. Cell cycle data was analyzed with FlowJo CL Software, using the Dean-Jett-Fox model on the living population.

**RNA expression analysis**

RNAseq was performed in primary samples from patients with relapsed ALL (n=224) on the Illumina (San Diego, USA) platform using the Illumina Stranded Total RNA Prep, Ligation with Ribo-Zero Plus protocol at Alacris Theranostics GmbH (Berlin, Germany). Expression values are in reads per kilobase per million mapped reads (RPKM), which corresponds approximately to the number of mRNA copies per cell. Robust Multichip Average (RMA) data from cell lines were obtained from The Genomics of Drug Sensitivity in Cancer Project^19^ (Affimetrix).

**CRISPR Dropout Screen**

To identify genetic dependencies to GSK-J4, we performed a CRISPR dropout screens in REH, NALM-6, and 697 cell lines expressing saCas9 (Addgene #52962). Lentiviral libraries coding for the sgRNA were built on the CROPseq-Guide-Puro plasmid (Addgene #86708) using the protocol described by Datlinger et al.^19^. Ten million cells per condition were seeded in triplicate in 8 mL of medium in 6-well plates and spinfected with the resulting lentiviral CRISPR library at a multiplicity of infection (MOI) of 0.3 for 1 hour at 800g in the presence of 4 μg/mL polybrene (H9268-5G, Sigma-Aldrich, St. Louis, United States). Twenty-four hours post-transduction, the medium was replaced with selection medium containing 1–2 μg/mL puromycin (A1113803, Thermo Scientific, United States) and GSK-J4 at the EC20 concentration (0.3µM for all cell lines).

Transduced cells were passaged every 3–4 days in the same selection medium, and 2 × 10⁶ cells were harvested and counted at days 3, 7, and 14 post-transduction. Genomic DNA was extracted from harvested cells using the Monarch Genomic DNA Purification Kit (T3010S, New England Biolabs, United States). sgRNA were amplified from the extracted DNA by PCR with the primer pair:

- Read2_U6 5' GTGACTGGAGTTCAGACGTGTGCTCTTCCGATCTGGGCCTATTTCCCATGATTCC 3'
- Read1_gRNAscaffhold 5' ACACTCTTTCCCTACACGACGCTCTTCCGATCTGACTCGGTGCCACTTTTTCA 3'.

The amplicons were size selected with AMPure XP beads (Beckman Coulter, Brea, CA) then barcoded into a sequencing library using:

- SI primers 5'AATGATACGGCGACCACCGAGATCTACACTCTTTCCCTACACGACGCTC 3'
- custom designed barcoding primers DOSCBC-XX 5' CAAGCAGAAGACGGCATACGAGAT-NNNNNN-GTGACTGGAGTTCAGACGTGT 3'

Libraries were sequenced with a target of 1.5M reads per condition (drug x timepoint x cell line) with MiSeq Reagent Kit v2 and NextSeq500 Mid Output v2.5 (Illumina)). Reads were aligned with bowtie2 2.5.0 (Langmead and Salzberg, 2012) on a synthetic genome containing the each spacer sequence flanked by the 250bp before and the 142bp after inserted by the CROPseq-Guide-Puro plasmid, and quantified with htseq-count 2.0.4 (Anders et al., 2015).

For analysis of sgRNA effect using beta scores, MAGeCK MLE v0.5.9.5 (Li et al., 2014) was employed. Here we used the model: measurement_Drug_dN = Drug_d3 + dN + Drug_dN where Drug_d3 is the library composition measured at d3, dN is the dropout effect at day N and Drug_dN is the enrichment due to the effect of the drug at day N. All results are presented for day 14.

**Protein quantification**

Western blot analysis was performed in cells lysed with RIPA buffer and following the protocol described with the Trans-Blot® Turbo™ Transfer System (Bio-Rad Laboratories GmbH, Munich, Germany). Horseradish peroxidase (HSP)-conjugated antibodies against CREB (4820S), CREBBP (7389S) and Tri-Methyl-Histone H3 (9733T) and used for western blotting were purchased from Cell Signaling (Danvers, USA). An HSP-conjugated antibody against HSP60 (GTX110089, Genetex, Irvine, USA) was used as a loading control. Antibodies were incubated overnight, diluted 1:1000. Bands were quantified using ImageJ 1.48v software (Rasband, W.S., ImageJ, U. S. National Institutes of Health, Bethesda, Maryland, USA, <https://imagej.nih.gov/ij/>, 1997-2018) by using the *Analyze>Gels>Plot Lanes* function.

**BH3 profiling**

Dynamic BH3 profiling of BCP-ALL cell lines was performed as previously described . After treatment for 16h with GSK-J4, cells were stained using the viability marker, Zombie Violet (423113, BioLegend, Koblenz, Germany), for 10 min at room temperature, then washed with PBS and resuspended in 330 µL of MEB buffer (150 nM mannitol, 10 mM HEPES-KOH pH 7.5, 150 mM KCl, 1 mM EGTA, 1 mM EDTA, 0.1% BSA and 5 mM succinate). In parallel, peptide solutions were prepared using MEB buffer with 0.002% of digitonin (D141, Sigma-Aldrich, St. Louis, United States) and 12 different peptide solutions with final concentrations of 25 µM alamethicin (BML-A150-0005, Enzo Life Sciences, Lörrach, Germany), 0.1 µM BAD BH3 peptide, 100 µM HRK BH3 peptide, 10 µM MS1 BH3 peptide and a DMSO only control. Cells in a volume of 25 µL were added to 25 µL of each peptide solution in a 96-well plate (3795, Corning, Madrid, Spain) and incubated at room temperature for 1 h. After incubation, cells were fixed with 25 µL 8% paraformaldehyde in PBS for 15 min and neutralized with 50 µL N2 buffer (1.7 M tris base, 1.25 M glycine at pH 9.1). Finally, 25 µL intracellular staining buffer (1% Tween20, 5% BSA in PBS) containing a 1:1,000 dilution of cytochrome C antibody (Alexa Fluor R647 anti-Cytochrome c—6H2.B4, 612310, BioLegend) was added, and plates were incubated overnight at 4°C. Results were analyzed using a Cyek Aurora cytometer (Cytek Aurora, Amsterdam, the Netherlands) and processed with FlowJo to quantify cytochrome c release (%priming). Δ%priming stands for the difference of %priming between non-treated and treated cells for each specific peptide. All results are represented as the mean of at least three independent experiments.

**Statistical analysis**

Statistical analyses were performed using Graphpad Software. In vitro drug synergy was analyzed using Combenefit Software v2.021 (University of Cambridge, Cambridge, UK). For the HSA model, the reference effect for the combination (a,b) is obtained by taking the greatest effect (lowest residual compared to control) between the two drugs as single agents:

The sum of the synergy scores incorporates a weight based on dose-response, which bias the total score towards synergy that achieves the highest effect. Hence, a synergy of 50% leading to a combined full effect (100%) will have more weight than if the corresponding effect was only 20 or 30%.

**Supplementary Figures**

**Supplementary Figure 1**

***Supplementary Figure 1. Correlation between CREBBP RNA expression and CREB1 or EP300.*** *RNAseq was performed in n=224 ALL patient samples and CREBBP expression (RPKM; Reads Per Kilobase per Million mapped reads) was inversely correlated to CREB1 (A) or directly correlated to EP300 (B). Red dots represent samples overexpressing CREBBP 19/224 (8.5% of cases). Pearson correlation was performed to obtain r and p values. Samples overexpressing CREBBP were identified by ordering the samples by their expression and finding the cutoff expression (Rc) in which the Jaccard distance in the log Expression – Sample expression Rank is maximized, providing a bias-free separation between low and high expressors of the genes (See Figure 1A).*

**Supplementary Figure 2**

***Supplementary Figure 2. Influence of GSK-J4 on cell cycle phases in BCP-ALL cell lines.*** *Cell cycle analysis of BCP-ALL cell lines (n=4) treated with GSK-J4 1µM for 48h, stained with CytoPhase, propidium iodide (PI), and analyzed via flow cytometry. A t-test comparing the G1 phase proportion between treated and untreated cells revealed no statistically significant differences. Data shows mean values of living cells (PI-) and SD of n=4 independent experiments.*

**Supplementary Figure 3**

***Supplementary Figure 3. Band intensity quantifications from Western Blot analysis of BCP-ALL cells treated with GSK-J4.*** *Data corresponds to the shown in figure 2A, where band intensity was normalized to loading control HSP60 and made relative to cells treated with vehicle (DMSO). Means and SD is shown for n=3-5 independent experiments for the cell lines and n=1 for the patient sample.*

**Supplementary Figure 4**

***Supplementary Figure 4. Correlation between sensitivity to GSK-J4 and dexamethasone, and CREBBP RNA and protein levels in ALL cell lines from the FORALL database.*** *(A) Selective drug sensitivity scores for GSK-J4 (sDSS) correlated with CREBBP RNA expression levels (n = 58 cell lines) and (B) CREBBP protein levels (n = 72 cell lines). (C) Selective drug sensitivity scores for dexamethasone (sDSS) correlated with CREBBP RNA expression levels (n = 58 cell lines) and (D) CREBBP protein levels (n = 72 cell lines). Figures were generated using the Functional Omics Resource of Acute Lymphoblastic Leukemia (FORALL) website and database^15^.*

**Supplementary tables**

| **Supplementary Table 1. Clinical characteristics of patients included in Figure 1G-H** | | | | | |
| --- | --- | --- | --- | --- | --- |
| **Patient** | **Disease state** | **Genetic Background** | **Response to induction treatment (MRD)** | **EC50 values *in vitro*** | **Relevant Mutations** |
| **1** | 1st relapse | *P2RY8::CRLF2* | Good response (MRD negative after induction) | 8,41 | *CREBBP, SETD2, AKT2, EP300* (*NF1* splice-acceptor variant), CHEK2 mutation (germline) |
| **2** | 2nd relapse | *ETV6::RUNX1* | Nonresponse; high MRD persistence during blinatumomab ; response to inotuzumab | 4,50 | *ETV6, WHSC1*; Deletions: *NR3C1, MHS2, IKZF1, PAX5* |
| **3** | 1st relapse | *SEPT9::ABL1* | day 28 after induction: 3E-03; good response to blinatumomab | 6,26 | *NOTCH1, FLT3, ABL1; NFKBIE, NOTCH2* |
| **4** | 1st relapse | *BCR::ABL1* | Nonresponse | 4,99 | *ABL1* |
| **5** | 2nd relapse | *ETV6::ABL1* | Nonresponse | 14,93 | +Chr.12, 8, 5; Chr.9, 5, MTOR, JAK1, PMS2; del IKZF1, del CDKN2B |
| **6** | 1st relapse | *BCR::ABL1* | Good response (MRD negative after induction) | 7,26 | *ABL1*, *MLH1* |
| **7** | 2nd relapse | *TCF3::PBX1* | Morphological Nonresponse; died after SCT | 3,28 | *TP53* (homozygote), *ATR* (homozygote) |
| **8** | 1st relapse | *BCR::ABL1* | Good response (MRD negative after induction and all following time points) | 1,76 | *POLE, FLT4*; Deletions: *IKZF1, CDKN2A/B* (both alleles), *PAX5, BTG1* |
| **9** | 1st relapse | *BCR::ABL1* | Good Response | 1,98 | *EP300* mut |
| **10** | 1st relapse | *SEPT9::ABL1* | Good response to blinatumomab | 1,64 | *NOTCH1, FLT3, ABL1; NFKBIE, NOTCH2* |
| **11** | 1st relapse | low hypodiploid | Good response (MRD after induction treatment: < 10-3 - =/> 10-4) | 5,73 | *TP53* mutation (germline) |
| **12** | 2nd relapse | *KMT2A::AFF1* | Only CD19 CAR T-cell treatment, MRD reappearance after 2 months | 4,96 | *KMT2A::AFF1* |
| **13** | 1st relapse | *P2RY8::CRLF2* | Poor response (MRD after induction treatment: < 10-2 - =/> 10-3) | 5,68 | P2RY8*::CRLF2* fusion, *IL7R* mutation, *PTPN11* mutation, *TP53* deletion, *CDKN2A* deletion, *PAX5* amplification |
|  |  |  |  |  |  |
|  |  |  |  |  |  |
| MRD (minimal residual disease), SCT (stem cell transplantation). | | | | | |

| **Supplementary Table 2. Summary of drug synergies in BCP-ALL cell lines** | | | | | | | |  |  |  | |  |
| --- | --- | --- | --- | --- | --- | --- | --- | --- | --- | --- | --- | --- |
|  |  |  |  |  | |  |  | | |  | |  |
|  |  |  | **Venetoclax** |  | |  | **Navitoclax** |  |  |  | |  |
| **Cell line** | **Genetic background** | ***CREBBP mutations**** | **No. of synergistic combinations**** | | **Max. synergy score**** | **Sum of synergy scores**** | **No. of synergistic combinations**** | **Max. synergy score**** | | | **Sum of synergy scores**** | |
| 697 | *TCF3-PBX1* |  | 8 | | 23,44 | 27,2 | 4 | 29,98 | | | 18,11 | |
| NALM-6 | *DUX4 rearranged* | c.5059T>C; c.4133+49G>A; c.4134-163C>T | 8 | | 65,1 | 75,82 | 8 | 56,65 | | | 53,72 | |
| REH | *ETV6-RUX1* | c.1942-1G>T | 1 | | 29,43 | 20,12 | 2 | 31,61 | | | 22,57 | |
| HAL01 | *TCF3-HLF* |  | 4 | | 38,95 | 35,75 | 3 | 43,87 | | | 33,49 | |
| *Mutation status obtained from COSMIC database^20^. | | | | | | | |  |  |  |  |  |
| **Data analyzed using Combenefit software. | | | | | | | |  |  | |  |  |
